# Soluble HLA-associated peptide from PSF1 has a cancer vaccine potency

**DOI:** 10.1038/s41598-017-11605-2

**Published:** 2017-09-11

**Authors:** Mari Yoshida, Yukichi Ishioka, Takamasa Ozawa, Hirohisa Okuyama, Motofumi Iguchi, Takeshi Ota, Takaomi Ito, Morio Nagira, Atsushi Morita, Hidekazu Tanaka, Hisamichi Naito, Hiroyasu Kidoya, Nobuyuki Takakura

**Affiliations:** 1 Biomarker R&D Department, Shionogi & Co., Ltd, Osaka, 561-0825 Japan; 2 Department of Signal Transduction, Research Institute for Microbial Diseases, Osaka University, 3-1 Yamada-oka, Suita, Osaka 565-0871, Osaka, 561-0825 Japan; 3 Drug Discovery & Disease Research Laboratory, Shionogi & Co., Ltd, Osaka, 561-0825 Japan; 4 Medical Affairs Department, Shionogi & Co., Ltd, Osaka, 530-0012 Japan; 5 Shionogi TechnoAdvance Research Co., Ltd, Osaka, 561-0825 Japan

## Abstract

Partner of sld five 1 (PSF1) is an evolutionary conserved DNA replication factor involved in DNA replication in lower species, which is strongly expressed in normal stem cell populations and progenitor cell populations. Recently, we have investigated PSF1 functions in cancer cells and found that PSF1 plays a significant role in tumour growth. These findings provide initial evidence for the potential of PSF1 as a therapeutic target. Here, we reveal that PSF1 contains an immunogenic epitope suitable for an antitumour vaccine. We analysed PSF1 peptides eluted from affinity-purified human leukocyte antigen (HLA) by mass spectrometry and identified PSF1_79–87_ peptide (YLYDRLLRI) that has the highest prediction score using an *in silico* algorithm. PSF1_79–87_ peptide induced PSF1-specific cytotoxic T lymphocyte responses such as the production of interferon-γ and cytotoxicity. Because PSF1 is expressed in cancer cell populations and highly expressed in cancer stem cell populations, these data suggest that vaccination with PSF1_79–87_ peptide may be a novel therapeutic strategy for cancer treatment.

## Introduction

Cancer immunotherapy facilitates the inherent immune system by recognising molecular entities expressed specifically on tumour cells, resulting in elimination of these cells. Cytotoxic T lymphocytes (CTLs) play a key role in attacking cancer cells. CTLs distinguish cancer cells from normal cells by cancer-specific antigens that are presented as human leukocyte antigen (HLA)-bound peptides on the cell surface^1, 2^. Despite recent advances in cancer immunotherapy, the overall efficacy of these approaches remains limited^3–8^. Cancer stem cells (CSCs) are thought to be relevant to tumour metastasis because of their resistance to chemotherapy and radiation therapy, and contribute to recurrence following treatment^9, 10^. Current immunotherapies primarily target tumour differentiation antigens. Accordingly, not targeting CSCs may be a significant factor in treatment failures, and novel therapeutic strategies are required to target CSCs.

Partner of sld five 1 (PSF1) is a component of a tetrameric complex termed GINS, which consists of SLD5, PSF1, PSF2, and PSF3, and is well conserved evolutionarily. Our previous reports suggested that PSF1 expression is essential for early embryogenesis, maintenance of the immature hematopoietic cell pool size, and acute bone marrow regeneration in mice^11, 12^. Loss of PSF1 leads to embryonic lethality around the implantation stage, which is caused by the inability of cells in the inner cell mass to proliferate^11^. In addition, PSF1 is not only expressed in immature cells, but also in cancer cells^13–15^. Moreover, we have reported that high expression of PSF1 is associated with CSC properties^16^, suggesting that PSF1 is a target molecule in CSCs and a candidate cancer-specific antigen.

Recently, significant technological improvements in genomics and proteomics along with supportive bioinformatics and *in silico* prediction tools have facilitated major breakthroughs in the delineation of the HLA peptidome that is the target of anti-cancer T cell responses^17^. We predicted the epitopes of PSF1 by three *in silico* algorithms, namely “BIMAS” (Bioinformatics and Molecular Analysis Section, NIH)^18^, “SYFPEITHI” (Biomedical Informatics, Heidelberg/Department of Immunology, University of Tübingen)^19^, and “NetMHC” (Center for Biological Sequence Analysis, Technical University of Denmark)^20^. In addition, we revealed the HLA peptidome by mass spectrometry (MS). MS is a methodology to comprehensively analyse the repertoire of the HLA peptidome presented naturally *in vivo*. Each having inherent advantages and disadvantages, both *in silico*-based prediction and direct MS-based approaches led to successful identification of the HLA peptidome for the development of cancer immunotherapy.

The goal of the present study was to identify PSF1-derived peptides presented by HLA using two methodologies, *in silico*-based prediction and an MS-based approach. In addition, we examined whether the identified peptide could induce CTL responses from peripheral blood mononuclear cells (PBMCs) of healthy donors. Our results suggest that PSF1 peptide generated *in vitro* shows the characteristics of an effective peptide vaccine against CSCs.

## Results

### Strategy for identification of major histocompatibility complex (MHC) class I-presented epitopes from PSF1

**Figure 1:**
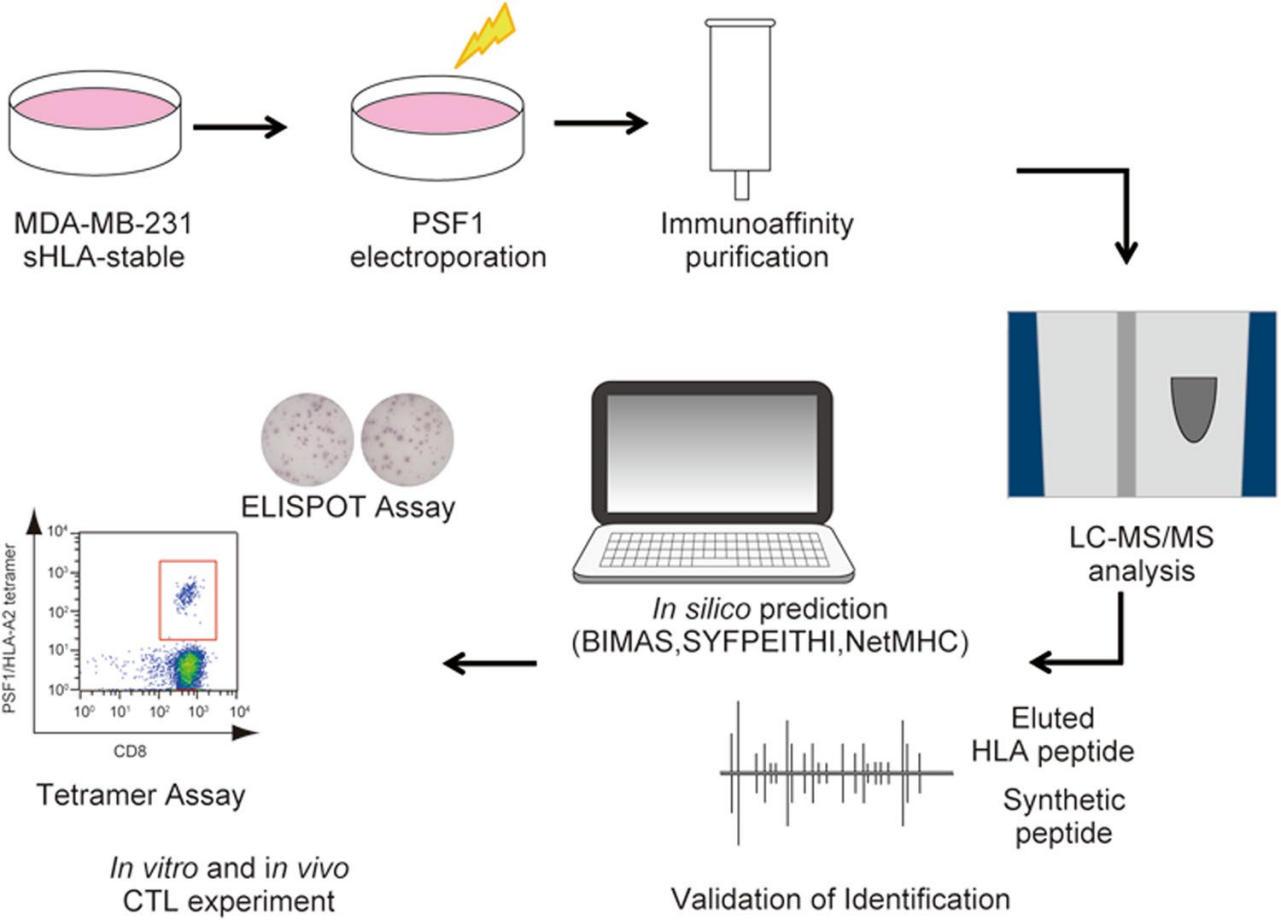
Immunoproteomics method work flow for identification and characterisation of PSF1-specific T cell epitopes. Strategy of antigen discovery by immunoproteomics for cancer immunotherapy. After immunoprecipitation of peptides by an anti-CII tag antibody column, sHLA-presented peptides were identified by tandem mass spectrometry. Adequacy of the identified peptides was further calculated by *in silico* prediction and validated by *in vitro* and *in vivo* immunogenic methods.

We identified processed and presented PSF1-specific, MHC class I-restricted epitopes using an immunoproteomics approach (Fig. 1). Briefly, we established the MDA-MB-231 cell line expressing soluble HLA-A*02:01 (sHLA) molecule (hereafter MDA-MB-231-sHLA cells) as reported previously^21^. PSF1 protein expression was observed in the original MDA-MB-231 cells; however, to identify PSF1-derived peptide captured on HLA effectively, we overexpressed the *PSF1* gene in MDA-MB-231-sHLA cells by transient transfection using the MaxCyte electroporation transfection system. To determine the optimal concentration of plasmid for transfection, cells were electroporated with varying amounts of plasmid. The protein expression level of PSF1 was increased several times (Supplementary Figure 1). Cell damage was observed in a dose-dependent manner (data not shown) and the optimal concentration was 125 µg/ml. Using this cell line, peptide/MHC complexes secreted in 3.6 L of culture medium were purified by an anti-19 amino acid collagen type II (CII) tag antibody^22^ column. To identify human PSF1-specific epitopes that formed a complex with MHC class I, transient expression of human PSF1 was induced in MDA-MB-231-sHLA cells.

**Figure 2:**
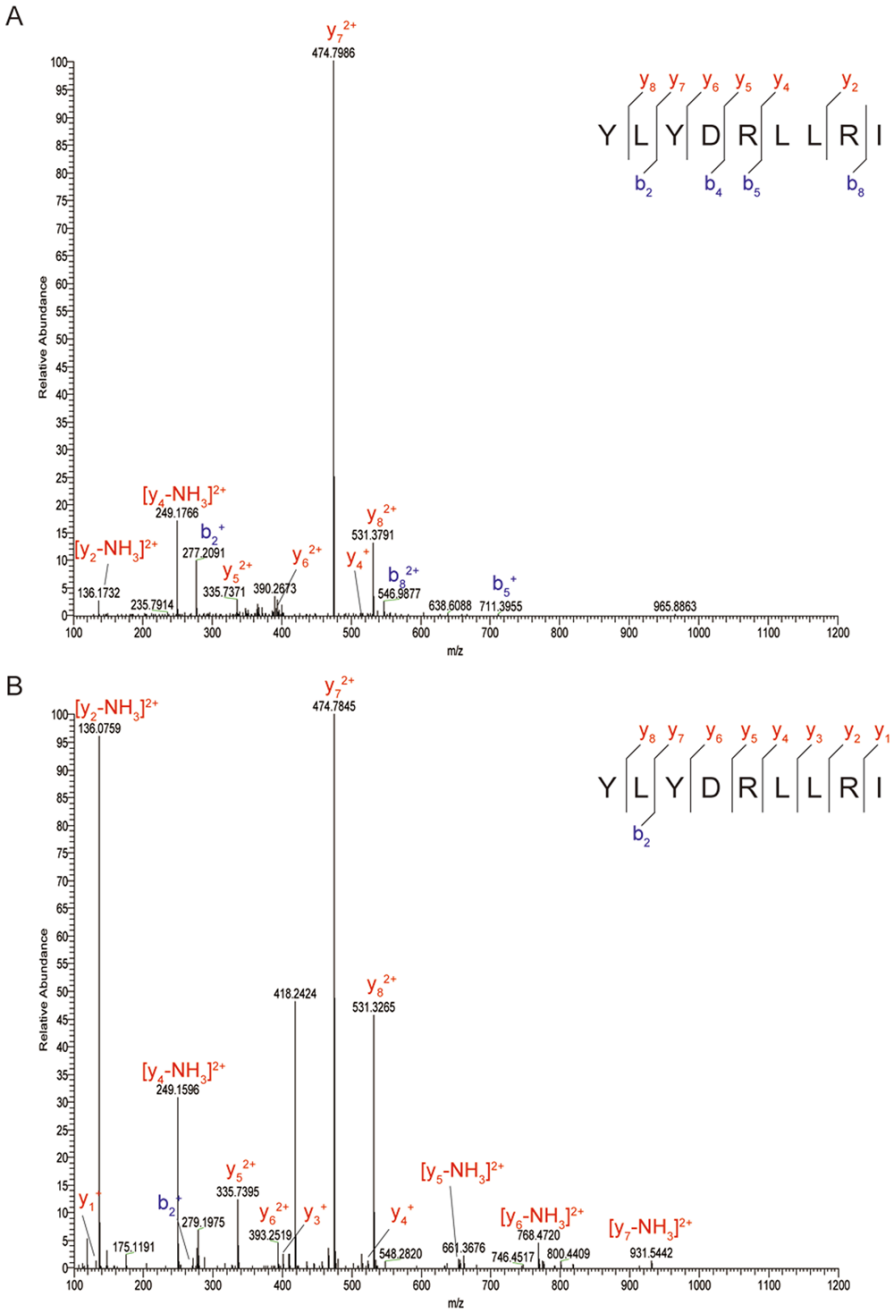
Product-ion spectra of tandem mass spectrometry. (**A**,**B**) Tandem mass spectra of sHLA-bound PSF1_79–87_ peptide derived from MDA-MB-231 cells (**A**) and the corresponding synthetic peptide (**B**). Spectral differences in endogenous and synthetic peptide spectra may be due to co-eluting peptides in the highly complex sample. Although present, not all fragment ions are labelled because of overlapping peak labels.

Subsequently, peptides bound to MHC class I molecules were isolated from the conditioned medium, and we identified YLYDRLLRI peptide (PSF1_79–87_ peptide) as an epitope derived from PSF1 by MS analysis (Fig. 2A). We then confirmed the sequence identity of the epitope using synthetic analogues. The MS/MS spectra of the endogenous peptide was in strong agreement with that of the synthetic peptide (Fig. 2B). In addition, we predicted the HLA-binding affinities of PSF1 peptides using three types of *in silico*-based software: BIMAS (http://www-bimas.cit.nih.gov/molbio/hla_bind/), SYFPEITHI (http://www.syfpeithi.de/), and

NetMHC (http://www.cbs.dtu.dk/services/NetMHC/). As shown in Table 1, PSF1_79–87_ peptide showed the highest score in all applied software (BIMAS and SYFPEITHI calculate binding scores, and NetMHC calculates IC_50_ values). We thought that it is of special value in PSF1_79–87_ peptide identified by two different methodologies: an MS-based experimental approach and *in silico*-based prediction. Additionally, PSF1_79–87_ peptide is a unique peptide compared with the other GINS component molecules (SLD5, PSF2, and PSF3).

**Table 1:** *In silico*-predicted HLA-A02-restricted 9-mer epitopes of PSF1.

Rank	BIMAS^a^		SYFPEITHI^b^		NetMHC^c^	
	Peptide sequence	Score	Peptide sequence	Score	Peptide sequence	Affinity
1	YLYDRLLRI	1448	YLYDRLLRI	27	YLYDRLLRI	3.7
2	QVLEEMKAL	65	SLATYMRSL	27	SLATYMRSL	45.2
3	SLYIEVRCL	31	SLYIEVRCL	27	KAMELIREL	272.6
4	FEVDDGTSV	26	KAMELIREL	25	SLYIEVRCL	768.0
5	SLATYMRSL	10	LLRNRRCTV	24	ALRWEYGSV	2347.6

^a^Website accessible at http://www-bimas.cit.nih.gov/molbio/hla_bind/. ^b^Website accessible at http://www.syfpeithi.de/. ^c^Website accessible at http://www.cbs.dtu.dk/services/NetMHC/. Peptide sequences are shown for the top five in each algorithm. BIMAS supplies a ranking according to the estimated half-life of the peptide/MHC α-chain/β_2_-microglobulin complex. SYFPEITHI provides scores based on the presence of certain amino acids in certain positions of the MHC class I-binding groove (anchor motifs). NetMHC (version 4.0) calculates the binding affinity of the peptide for selected HLA molecules, which is expressed as the predicted IC_50_ in nanomoles.

### Vaccination induces PSF1-specific CD8^+^ T cell responses

**Figure 3:**
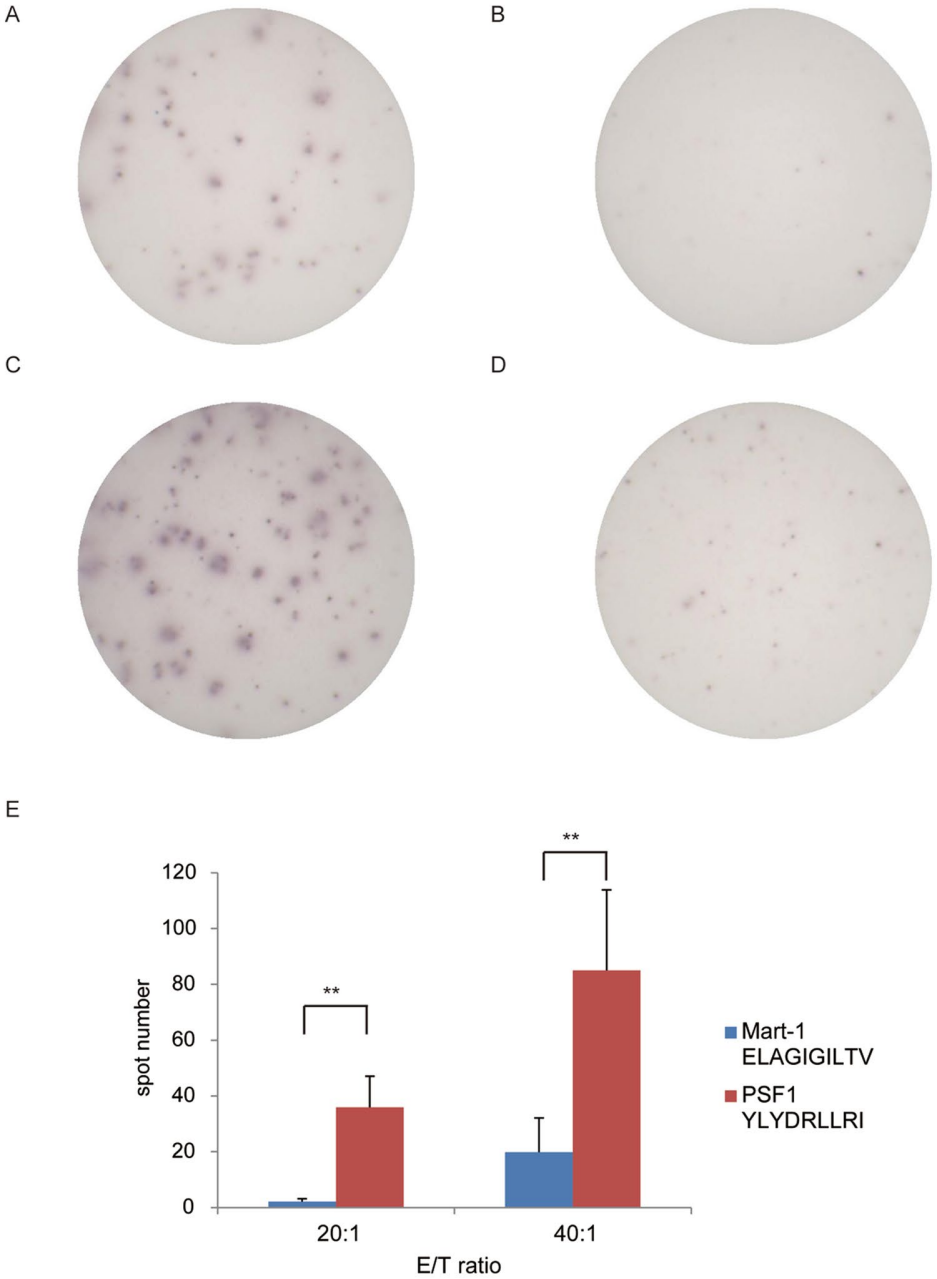
IFN-γ ELISPOT analysis of CD8^+^ T cells derived from PSF1 peptide-immunised CB6F1-Tg mice. (**A**–**D**) Representative images of PSF1_79–87_ peptide-specific spots. (**A**) E/T ratio of 20:1 with PSF1_79–87_ peptide, (**B**) E/T ratio of 20:1 with Mart-1_26–35_ peptide, (**C**) E/T ratio of 40:1 with PSF1_79–87_ peptide, and (**D**) E/T ratio of 40:1 with Mart-1_26–35_ peptide. (**E**) Quantitative evaluation of the number of specific spots at each E/T ratio. Data are the mean ± s.e.m. **P < 0.01. Significance was calculated by the Student’s t-test. N = 4 biological replicates.

To assess whether the experimentally identified peptide stimulates splenocytes *in vivo*, we used CB6F1-Tg (HLA-A*02:01/H2-Kb) (hereafter HLA-A*02:01 transgenic) mice. The HLA-A*02:01 transgenic mice were immunised with PSF1_79–87_ peptide in Montanide ISA 51VG to induce CTL activity. Two weeks after immunisation, splenocytes were harvested and co-cultured with T2 cells pulsed with PSF1_79–87_ peptide or Mart-1_26–35_ peptide as a control in an interferon (IFN)-γ enzyme-linked immunospot (ELISPOT) assay. We found that splenocytes produced a significant amount of IFN-γ in a peptide-specific manner only when co-cultured with T2 cells pulsed with PSF1_79–87_ peptide (Fig. 3A–E). At an effector cell/target cell (E/T) ratio of 20:1, these splenocytes showed 36 spot counts/well in response to T2 cells pulsed with PSF1_79–87_ peptide, whereas they showed 2 spot counts/well in the presence of T2 cells with negative-control peptide loading (Fig. 3E, *P* < 0.01). The trend was also observed significantly at an E/T ratio of 40:1. These observations strongly demonstrate the potential of PSF1_79–87_ peptide to be presented by HLA-A*02:01.

### Cytotoxic activity of PSF1_79–87_ peptide-reactive CTLs from PBMCs of HLA-A2-positive healthy donors

**Figure 4:**
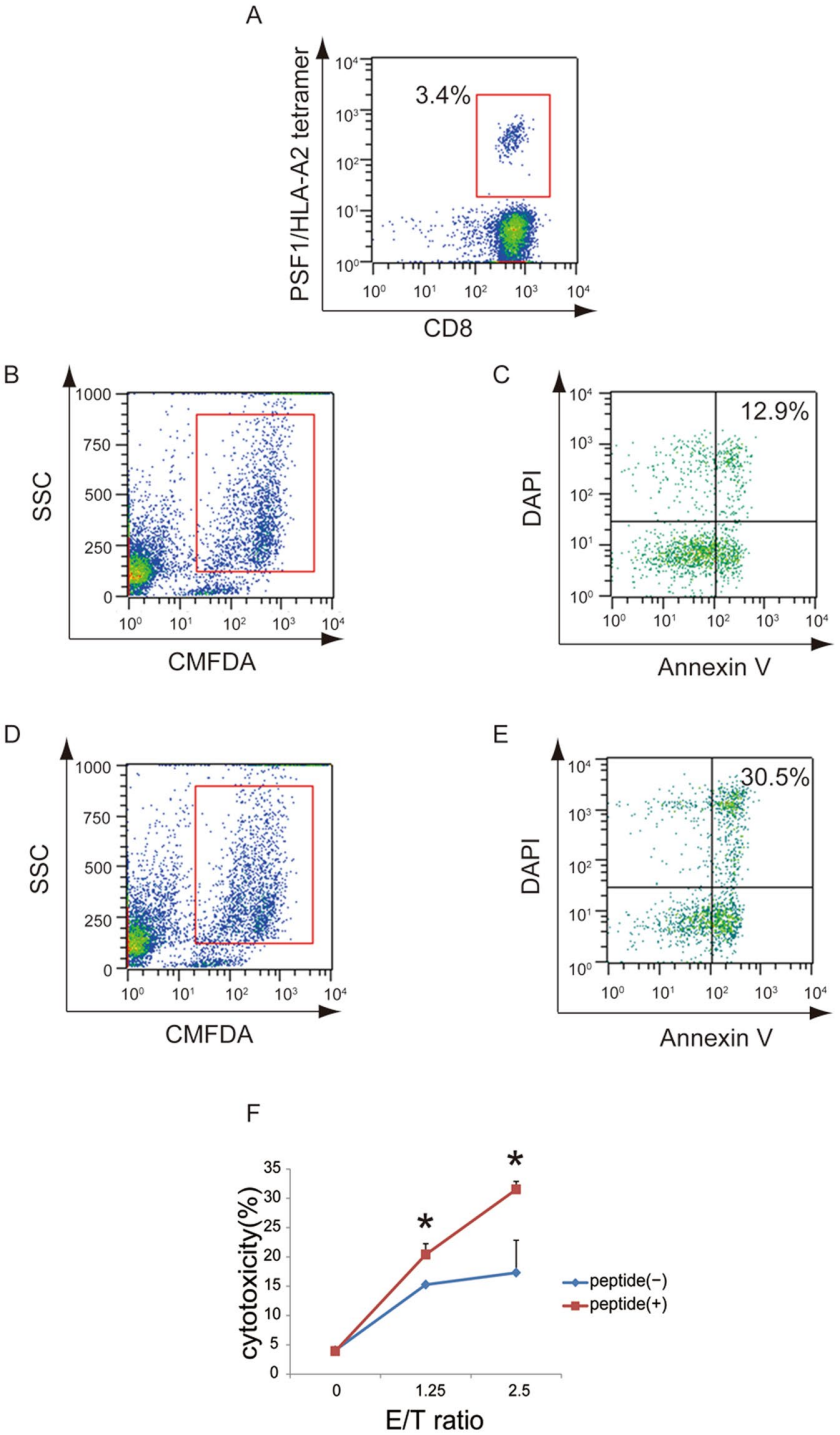
Cytotoxicity assays of PSF1 peptide. (**A**) Tetramer staining of PSF1-specific CTLs. CTLs obtained by incubation were subjected to double staining with a PE-labelled tetramer and APC-labelled anti-CD8 antibody. Fluorescent intensities were measured with a MACSQuant. (**B**,**C**) Plots are gated (red box) on the CMFDA-labelled target cells at an E/T ratio of 2.5 without PSF1_79–87_ peptide. The CMFDA-labelled target cells were sorted according to DAPI and Annexin V staining. (**D**,**E**) E/T ratio of 2.5 with PSF1_79–87_ peptide. (**F**) The cytotoxic rate of target cells was indicating by Annexin V and DAPI double-positive staining. PSF1_79–87_ peptide-pulsed cells (peptide+) or unpulsed cells (peptide−) obtained from CTLs specific for PSF1_79–87_ peptide (E; effector cell) and T2 cells (T; target cell) were co-cultured at E/T ratios of 1.25 and 2.5. Data are the mean ± s.e.m. *P < 0.05.

As a next step to validate the potency of PSF1_79–87_ peptide in the human immune system, we evaluated the peptide-specific immune responses of human CTLs. CTLs were generated by peptide stimulation of PBMCs isolated from healthy donors who were positive for HLA-A*02:01, and CD8^+^ T cells purified from PBMCs were co-cultured with monocyte-derived dendritic cells (MoDCs) pulsed with the peptide. To detect HLA-A*02:01-restricted and PSF1_79–87_-specific CTLs, we used a HLA-A*02:01-PSF1_79–87_ peptide tetramer and observed the population of HLA-A*02:01/PSF1_79–87_ peptide-tetramer^+^/CD8^+^ T cells (Fig. 4A).

To determine the functional cytotoxic activity of the peptide-specific CTLs, we tested their ability to kill T2 cells pulsed with PSF1_79–87_ peptide. The peptide-specific CTL line killed 30.5% of PSF1_79–87_ peptide-pulsed T2 cells and 12.9% of non-pulsed T2 cells (Fig. 4B–E). The cytotoxicity of CTLs was induced in a dose-dependent manner of the E/T cell ratio (Fig. 4F). These results suggest that vaccination with PSF1_79–87_ peptide could induce immune responses in the clinical setting.

## Discussion

CSCs mediate tumour metastasis by their relative resistance to current therapies and contribute to relapse following treatment^9, 10^. Many strategies that are not selective enough against CSCs can be toxic to healthy tissues, and patients are usually at risk of recurrence and metastasis because of the lack of CSC elimination^23^. Additionally, CSC populations are more resistant to conventional cancer therapies than non-CSC populations^24–26^. It was recently reported that CSCs might be immunogenic in certain settings. Thus, they have the potential to stimulate specific immune responses that can target and remove tumour CSCs in cancer patients^27^. We believe that novel therapeutic strategies by vaccination selectively targeting CSCs may be of the greatest therapeutic benefit. Our previous study suggested that PSF1 is highly expressed by malignant cancer cells, which are defined as CSCs, and that this molecule has important roles in the survival and proliferation of cancer cells^16^. Here, we identified PSF1-derived peptides presented by HLA by direct (MS-based) and indirect (*in silico*-based) methods (Fig. 1).

Considering that the class I HLA molecule is a cell surface protein with intracellular peptide epitopes, many indirect methods have been used to discover HLA-associated tumour rejection antigens. A small number of tumour antigens were revealed by purification of HLA-associated peptides from cell lysates. Thus far, identification of HLA-binding peptides from cell lysates has an innate limitation associated with detergent in the MS-based approach. To overcome the problem, we used secreted class I HLA molecules for peptide identification without detergent lysis^28^. In this study, we purified peptides associated with sHLA from conditioned medium of the MDA-MB-231 cell line expressing sHLA and human PSF1, and identified PSF1_79–87_ peptide by liquid chromatography coupled with tandem mass spectrometry (LC-MS/MS). We identified other proteins, such as histone, cytoskeletal proteins (actin and tubulin), and cytosolic chaperones (heat shock proteins). However we found no other cancer vaccine target proteins other than PSF1.

Comprehensive lists of peptide sequences predicted to associate with HLA can be obtained by *in silico* algorithms. It is one of the most powerful tools to reveal the peptidome that represents HLA-binding peptides. However, because of the intrinsic and unavoidable high number of false positives among the predicted epitopes, and the fact that these algorithms predict binding affinity and not cellular processing or immunogenicity^29^, supporting *in vitro* experiments are required to screen for the actual presented and immunogenic peptides. We carried out a preliminary analysis to compare CTL activity with binding affinity to HLA *in silico* in a mouse model, and found that it is difficult to predict the relationship between them. In the present study, we used a bioinformatics approach based on BIMAS, SYFPEITHI, and NetMHC algorithms to predict HLA-binding peptides derived from PSF1 (Table 1). Remarkably, the results obtained by MS-based analysis were confirmed by all *in silico* analyses. Currently, MS-based methods continue to drive significant improvements in sensitivity and throughput for discovery of HLA-binding peptides. We believe that, as larger data sets become available, predictive algorithms will be used to ensure the potential immunological significance of directly identified HLA ligands for further study, as shown in this report.

Several studies have reported the use of molecules expressed in CSCs as sources of antigens for vaccine development^30–33^. However, there are still few peptide vaccine targets in CSCs. For example, survivin, which is the smallest member of the inhibitor of apoptosis family of proteins, is a known marker of CSCs and important target for cancer vaccines and therapeutics^34^. Therapeutic outcomes using survivin as a vaccine have not been clarified in terms of killing CSCs clinically. In this study, we determined whether injection of PSF1_79–87_ peptide would cause immunological responses *in vivo*. We used HLA-A*02:01 transgenic mice to induce CTL activity. HLA-A*02:01 transgenic mice, which express a chimeric gene consisting of the α1 and α2 domains of human HLA-A*02:01 and the α3 domain of murine H-2K^b^, have been reported to be a versatile animal model for preclinical evaluation of peptide-based immunotherapy^35–37^. In our analysis, we verified immunological responses induced by PSF1_79–87_ peptide using this mouse model. Moreover, we confirmed that the CTLs induced by peptide-loaded MoDCs exerted a significant cytotoxic effect against PSF1_79–87_ peptide-expressing cancer cells. Although the population was not very large, these CTLs significantly killed T2 cells pulsed with PSF1_79–87_ peptide. Therefore, we believe that these CTL responses were effective and specific to PSF1_79–87_ peptide, and it is possible that PSF1_79–87_ peptide can target highly PSF1-expressing malignant cancer cell populations, i.e., CSCs. PSF1 is expressed in immature cell populations and stem cell populations [e.g., epiblasts during embryogenesis, bone marrow hematopoietic stem cell populations, sperm stem cells (spermatogonia)] in mice^11, 12^. However, the PSF1 expression levels of these normal cells are extremely lower compared with cancer cells. Moreover, it is not known whether the PSF1_79–87_ peptide binds to HLA expressed on normal stem cells. It will be necessary to compare captured PSF1_79–87_ peptide levels in normal stem cells and cancer stem cells *in vivo* in tumours in future research.

In summary, we identified a candidate cancer vaccine peptide for CSCs, PSF1_79–87_ peptide, derived from PSF1. We demonstrated that PSF1_79–87_ peptide induces CTL responses *in vitro* and *in vivo* in an HLA-A*02:01-restricted manner. This report is the first to reveal presented PSF1-specific T cell epitopes identified by direct (MS) and indirect (*in silico*) methods. Vaccination with the peptide identified in this study may provide a new cancer immunotherapy for targeting CSCs.

## Methods

### Animals

Nine-week-old female HLA-A*02:01 transgenic mice were purchased from Taconic Biosciences, Inc. (strain HLA-A*02:01, CB6F1-Tg (HLA-A*02:01/H2-Kb); Hudson, NY). All procedures were approved by the animal care and use committee of Shionogi & Co., Ltd. All experiments were performed in accordance with the Committee’s guidelines.

### Tissue culture

Human breast cancer cell line MDA-MB-231 obtained from the American Tissue Culture Collection (Manassas, VA) was transfected with a plasmid construct consisting of the expression vector pcDNA3.1(+) encoding sHLA with a CII tag (GEPGDDGPSGAEGPPGPQG) for purification^28, 38^. Stable transfectants were established using Lipofectamine LTX (Thermo Fisher Scientific, Waltham, MA) and selected with G418 (Thermo Fisher Scientific). Transient expression of PSF1 in sHLA-expressing MDA-MB-231 cells was induced by transfection with a plasmid construct consisting of the expression vector pcDNA3.3 encoding human PSF1 using the MaxCyte STX® Scalable Transfection System (MaxCyte, Gaithersburg, MD)

### Prediction of HLA-A*02:01-presented immunogenic 9-mer peptides

The PSF1 sequence was subjected to analysis by computerised HLA-binding prediction based on freely accessible online databases, BIMAS, SYFPEITHI, and NetMHC. All programs provide peptide sequences that are likely to be presented by the selected HLA molecules together with a ranking, score, or affinity. The top five peptides in each analysis were chosen.

### Purification and identification of PSF1 epitope peptides

Peptide-HLA-A*02:01 complexes were purified according to a previous report^39^ with minor modifications. A total of 3.6 L of collected culture supernatant (1.2 × 10^9^ cells) was pre-cleared with inactivated AminoLink Plus Coupling Resin (Thermo Fisher Scientific) to remove nonspecific proteins, followed by immunoprecipitation with anti-CII mouse monoclonal antibody (6G4)-immobilised AminoLink Plus Coupling Resin (Thermo Fisher Scientific). After a series of wash steps, the interacted epitope peptides were dissociated from HLA molecules using 10% acetic acid at 90 °C for 5 min and then passed through a 10 kDa molecular mass cutoff filter (Millipore Corp, Bedford, MA). The isolated peptides were fractionated using a MonoSpin SCX spin column (GL Sciences Inc., Tokyo, Japan) according the manufacturer’s instructions. The eluate from 350 mM NaCl was desalted using a MonoSpin C18 spin column (GL Sciences Inc.) according to the manufacturer’s instructions and dried using a savant SPD2010 SpeedVac system (Thermo Fisher Scientific).

### LC-MS/MS analysis of HLA-binding peptides

LC-MS/MS measurements were performed using an FTICR-MS combined with a nanoLC system (Paradigm MS4; Michrom Bioresources, Auburn, CA) and LTQ Orbitrap XL (Thermo Fisher Scientific). The prepared peptide samples were loaded onto a reverse-phase C18 trap column (L-column Micro 300 µm i.d. ×50 mm, 5 µm particles, 120 Å pore size; Chemicals Evaluation and Research Institute, Tokyo, Japan). The separation was carried out using a reverse-phase C18 analytical column (L-column Micro 100 µm i.d. ×150 mm, 3 µm particles, 120 Å pore size; Chemicals Evaluation and Research Institute) at a flow rate of 300 nl/min with solvent A (0.1% formic acid and 2% acetonitrile) and solvent B (0.1% formic acid and 90% acetonitrile) with the following linear gradient B%: 0–100 min, 5–45%; 100–100.5 min, 45–95%; 100.5–104.5 min, 95%; 104.5–105 min, 95–5%; 105–120 min, 5%. Collision-induced dissociation was performed for the three most intense precursor ions selected from each full scan in the Orbitrap (350–900 m/z; resolving power: 30000). PSF1_79–87_ synthetic peptide (Sigma-Aldrich, Tokyo, Japan) was analysed on an FTICR-MS combined with a nanoLC system (Easy-nLC 1000 system; Thermo Fisher Scientific) and Q Exactive bench-top quadrupole-orbitrap mass spectrometer (Thermo Fisher Scientific). PSF1_79–87_ synthetic peptides were loaded onto a reverse-phase C18 trap column (100 µm i.d. ×20 mm, 5 µm particles, 100 Å pore size; Thermo Fisher Scientific). The separation was carried out using a reverse-phase C18 analytical column (75 µm i.d. ×100 mm, 3 µm particles; Nikyo technos, Tokyo, Japan) at a flow rate of 300 nl/min with solvent A (0.1% formic acid) and solvent B (0.1% formic acid and 99.9% acetonitrile) with the following linear gradient B%: 0–10 min, 0–35%; 10–12 min, 35–90%; 12–20 min, 90%. Collision-induced dissociation was performed for the 10 most intense precursor ions selected from each full scan in the Orbitrap (350–900 m/z; resolving power: 17500).

### MS data analysis

Tandem mass spectral results were submitted to protein database searching using Mascot algorithm version 2.5.1 (MatrixScience, London, UK) via proteome discoverer version 1.3 (Thermo Fisher Scientific) against the Uniprot human proteome database without isoforms (Proteome ID: UP000005640; download date: 29-Jan-2016) containing 69986 entries. The data were searched with no enzyme specificity, methionine oxidation (115.995 Da), and a 10 ppm precursor ion mass tolerance. Fragment ion mass tolerance was specified at 0.4 Da or 0.02 Da for MS/MS data acquired by the LTQ or Orbitrap, respectively. MS/MS spectra from synthetic peptides were compared with the spectrum from the eluted natural peptide to prove the identity^40^.

### Peptide immunisation and collection of epitope-specific CTLs *in vivo*

HLA-A*02:01 transgenic mice were immunised with 50 µg PSF1_79–87_ peptide in PBS plus Montanide ISA 51VG (Seppic Inc, Fairfield, NJ) (50:50 emulsion) subcutaneously. Two weeks after immunisation, the spleens were passed through a 70-µm cell strainer (BD Biosciences, San Jose, CA) using a sterile 5 ml syringe plunger. The splenocytes were lysed with BD Pharm Lyse Buffer (BD Biosciences) to eliminate red blood cells, washed and re-suspended in ELISPOT assay medium (RPMI 1640: AIM-V = 1:1) containing 10% FBS (Thermo Fisher Scientific), 55 µM 2-mercaptoethanol (Thermo Fisher Scientific), 1 mM sodium pyruvate (Thermo Fisher Scientific), 1% MEM non-essential amino acids solution (Thermo Fisher Scientific), 1% penicillin-streptomycin (Thermo Fisher Scientific). The cells were used as the effector cells for an ELISPOT assay^41^.

### ELISPOT assay

T2 cells (ATCC CRL-1992; American Type Culture Collection) were pre-incubated with PSF1_79–87_ peptide (30 µg/ml) or negative control peptide (Mart-1_26–35_) in AIM-V culture medium (Invitrogen, Carlsbad, CA) overnight and irradiated with X rays (30 Gy). Antigen-stimulated IFN-γ release as a measure of CTL activation was assayed using a Mouse IFN-γ ELISpotPLUS (MABTECH, Stockholm, Sweden) according to the manufacturer’s instructions. In brief, 5 × 10^4^ target cells per well were co-cultured with effector cells at an E/T ratio of 40:1 or 20:1 in replicate wells overnight. Spots were quantitated using an ImmunoSpot (R) S5 Versa Analyzer (Cellular Technology Ltd, Cleveland, OH)^42, 43^.

### Induction of PSF1-reactive human CTLs

Blood was obtained from HLA-typed healthy volunteers^44^. All volunteers provided informed consent that was approved by the Ethics Committee of Shionogi & Co., Ltd. All procedures were approved by the Committee and all experiments were performed in accordance with the Committee’s guidelines. MoDCs were generated by *in vitro* culture as described previously^45, 46^. Briefly, PBMCs isolated from healthy volunteers positive for HLA-A*02:01 using Ficoll-Plaque solution (GE Healthcare UK, Buckinghamshire, UK). CD8^+^ or CD14^+^ cell populations were purified from PBMCs with microbeads (Miltenyi Biotec, Bergisch Gladbach, Germany). CD14^+^ cells were subjected to differentiation induction to MoDCs, whereas CD8^+^ cells were frozen for storage. CD14^+^ cell population was cultured in the presence of 100 ng/ml granulocyte-macrophage colony-stimulating factor (R&D Systems Inc., Minneapolis, MN) and 10 ng/ml interleukin (IL)-4 (R&D Systems) in AIM-V culture medium containing 2% heat-inactivated autologous plasma in two RepCell plates (CellSeed Inc., Tokyo, Japan). After 5 days of culture, 0.1 KE/ml OK-432 (Chugai Pharmaceutical Co., Ltd., Tokyo, Japan) was added to one plate and pulsed with 20 µg/ml PSF1_79–87_ peptide. On day 6, these peptide-pulsed MoDCs were irradiated with X rays (30 Gy) and mixed with thawed CD8^+^ T cells. These cultures were prepared in 96-well plates with each well containing 1 × 10^4^ peptide-pulsed MoDCs, 1 × 10^5^ CD8^+^ T cells, 20 U/ml IL-2 (Shionogi & Co., Ltd., Osaka, Japan) and 10 ng/ml IL-7 (R&D Systems) with 2% autologous plasma. On day 12, the remaining CD8^+^ T cells were stimulated with peptide-pulsed autologous MoDCs. We prepared MoDCs each time in the same manner as described above. On day 18, CD8^+^ T cells were treated with 100 ng/ml IL-15 (Miltenyi Biotec). The antigen-specific responses of the CTLs were investigated using a cytotoxicity assay on day 26^47^.

### Staining of HLA-A*02:01-restricted PSF1_79–87_ CTLs

Cells were incubated with a phycoerythrin (PE)-labelled HLA-A*02:01 PSF1_79–87_ peptide-binding HLA tetramer (PSF1 tetramer) (Medical & Biological Laboratories, Nagoya, Japan) and then stained with an allophycocyanin (APC)-labelled anti-human CD8 monoclonal antibody (BD Biosciences). The cells were analysed by a MACSQuant device (Miltenyi Biotec). HLA-A*02:01-restricted PSF1_79–87_ CTLs were defined as CD8^+^/PSF1-tetramer^+^ T cells^48^.

### Cytotoxicity assay

CTLs derived from PBMCs were co-cultured with peptide-pulsed/unpulsed T2 cells at several E/T ratios. In brief, T2 cells were pulsed with 20 µg/ml PSF1_79–87_ peptide overnight at 37 °C in AIM-V culture medium. As target cells for use in the cytotoxicity assay, 1 µM CellTracker® Green 5-chloromethylfluorescein diacetate (CMFDA) (Invitrogen) was added to T2 cells, and then these cells were allowed to react with each other at 37 °C for 15 min. The labelled target cells were rinsed three times, seeded at 1 × 10^4^ cells per well in a 96-well half area plate (Corning, Tokyo, Japan), and cultured with PSF1_79–87_ CTLs in a final volume of 200 µl. After 5 h of incubation, the cells were subjected to an Annexin V Apoptosis Detection Kit APC (eBioscience, San Diego, USA) at room temperature for 15 min in the dark and then immediately analysed by the MACSQuant. Cellstain DAPI solution (0.1 µg/ml; Dojindo Laboratories, Kumamoto, Japan) was added to distinguish live or dead cells. To quantify apoptotic target cells, the percentages of CMFDA-positive and Annexin V/DAPI double-positive cells were calculated^49^.

## Electronic supplementary material

Supplementary Information
