# Supplementary material for: Soluble HLA-associated peptide from PSF1 has a cancer vaccine potency
Source: Sci Rep. 2017 Sep 11;7:11137. doi: 10.1038/s41598-017-11605-2 (PMC5593935; doi:10.1038/s41598-017-11605-2)
Supplement: Supplementary file 1 — Supplementary Information [file 41598_2017_11605_MOESM1_ESM.pdf]

Supplementary Information

Soluble HLA-associated peptide from PSF1 has a cancer vaccine potency

Mari Yoshida[1,2], Yukichi Ishioka[3], Takamasa Ozawa[3], Hirohisa Okuyama[3], Motofumi Iguchi[4], Takeshi Ota[3], Takaomi Ito[5], Morio Nagira[3], Atsushi Morita[1], Hidekazu Tanaka[3], Hisamichi Naito[2], Hiroyasu Kidoya[2], Nobuyuki Takakura[2]*

[1]Biomarker R&D Department, Shionogi & Co., Ltd, Osaka 561-0825, Japan

[2]Department of Signal Transduction, Research Institute for Microbial Diseases, Osaka University, 3-1 Yamada-oka, Suita, Osaka 565-0871, Osaka 561-0825, Japan

[3]Drug Discovery & Disease Research Laboratory, Shionogi & Co., Ltd, Osaka, 561-0825, Japan

[4]Medical Affairs Department, Shionogi & Co., Ltd, Osaka 530-0012, Japan

[5]Shionogi TechnoAdvance Research Co., Ltd, Osaka 561-0825, Japan

Supplementary Figure S1.

Western blot analysis of PSF1 expression in MDA-MB-231 cells

MDA-MB-231 cells were lysed in lysis buffer consisting of 50 mM Tris-HCl (pH 7.5), 150 mM NaCl, 1% NP-40, 0.5% NaDoc, 0.1% SDS and protease inhibitor (Roche Applied Science, Indianapolis, IN). The protein concentration of the lysate was measured using a BCA Assay kit (Thermo Fisher Scientific). The cell lysate containing 10 μg protein was subjected to SDS-PAGE, transferred to a polyvinylidene difluoride membrane and immunoblotted using primary antibodies for PSF1 (clone Aho57.2; GeneStem, Osaka Japan) and β-actin (clone AC-15; Sigma-Aldrich), followed by horseradish peroxidase-conjugated anti-rat IgG (Thermo Fisher Scientific) or anti-mouse IgG (GE Healthcare) secondary antibodies. The blots were visualized using chemiluminescence with ECL (GE Healthcare).

(A) Western blot analysis of tissue lysates from MDA-MB-231-sHLA (Mock) cells transfected with varying amounts of DNA (100, 150 and 200 μg/ml). (B) Bar graph for PSF1 expression quantified by densitometry and expressed as the fold change from Mock cells. The densitometric data were normalized by β-actin.

A

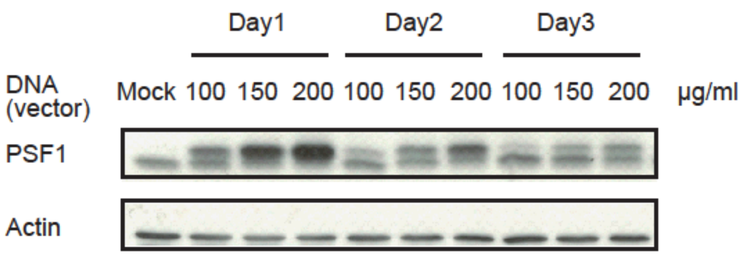
Day1
Day2
Day3
DNA (vector)
Mock 100 150 200 100 150 200 100 150 200 μg/ml
PSF1
Actin

B

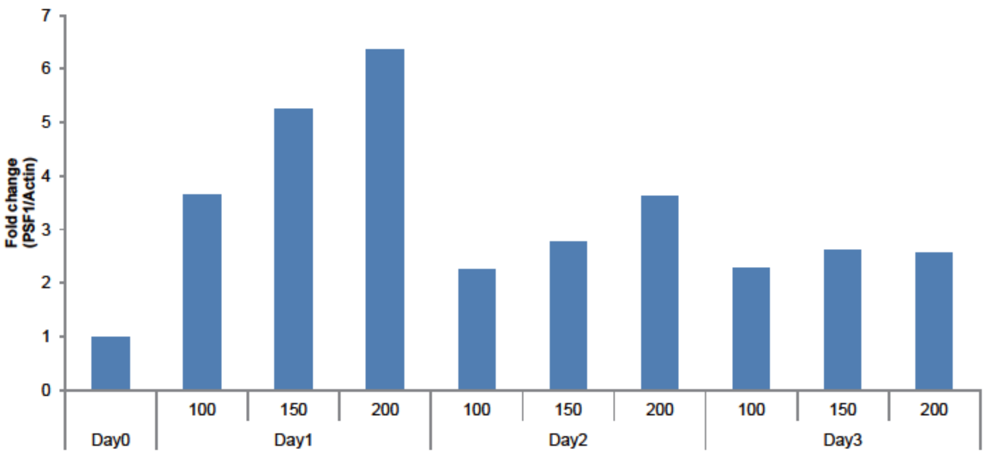
Fold change (PSF1/Actin)
0
1
2
3
4
5
6
7
100
150
200
100
150
200
100
150
200
Day0
Day1
Day2
Day3
